# Long-term growth and neurodevelopment of extremely preterm infants randomized to fortified human milk diets enriched with a protein supplement

**DOI:** 10.1038/s41390-025-04354-w

**Published:** 2025-08-22

**Authors:** Seabrook Jeffcoat, Ariel A. Salas

**Affiliations:** Division of Neonatology, University of Alabama at Birmingham, Birmingham, AL USA

## Abstract

**Background:**

Observational studies indicate that higher protein intake can promote long-term growth and reduce the risk of neurodevelopmental impairment (NDI) in extremely preterm infants fed human milk.

**Methods:**

This was a secondary analysis of a randomized trial in which 56 preterm infants born at 25–28 weeks of gestation were randomly assigned to receive either standard fortified milk or fortified milk supplemented with extensively hydrolyzed protein after postnatal day 14. The primary outcome of this analysis was moderate to severe NDI or death at approximately 2 years of corrected age. Anthropometric outcomes at 2 years of corrected age were also analyzed.

**Results:**

The outcome of moderate to severe NDI or death was assessed in 52 infants and did not differ between groups (42% vs. 43%). Bayley assessments were completed in 33 eligible infants (61%). Cognitive, language, and motor scores did not differ between groups. Similarly, weight (*n* = 35), length (*n* = 30), head circumference (*n* = 28) and their respective z-scores at approximately 2 years of corrected age did not differ between groups.

**Conclusion:**

In this analysis with lower-than-expected follow-up rates, a protein-enriched fortified milk diet neither reduced the risk of moderate to severe NDI nor promoted long-term growth.

**Impact:**

- Protein-enriched fortified milk diets after the first 2 postnatal weeks do not appear to significantly affect long-term growth or the likelihood of surviving without neurodevelopmental impairment in infants born extremely preterm.
- Further research is needed to determine the optimal timing for introducing protein-enriched fortified milk diets.
- More randomized clinical trials with extended follow-up data are essential to provide evidence-based guidance for clinical practice.
  **Category of Study**: Clinical

## Introduction

Extremely preterm (EPT) infants born before 28 weeks of gestation account for a small fraction of all live births (<1%),^1,2^ but face the highest risk of mortality and disability.^3^ Survival rates among EPT infants receiving proactive life support range from 24% for those born at 22 weeks to 90% for those born at 27 weeks. Similarly, the chances of surviving without any neurodevelopmental impairment (NDI), as measured by the Bayley Scales of Infant Development (BSID), are low among survivors born at 22 weeks (23%) and higher among survivors born at 27 weeks (71%).^4^ At 2 years of age, 29.3% of EPT infants exhibit moderate NDI, and 21.2% suffer from severe NDI.^5^ These neurodevelopmental risks are higher in the nearly half of EPT infants who experience postnatal growth faltering.^6,7^

Nutritional interventions, particularly increased enteral protein intake, have the potential to improve both growth and neurodevelopmental outcomes in this population. Enteral protein supplementation has been associated with a lower risk of postnatal growth faltering at 36 weeks postmenstrual age (PMA) and 24 months corrected age (CA)^8^ and improvements in postnatal weight, length, and head circumference.^9^ Moreover, higher growth velocities from birth to discharge have been associated with a lower incidence of prematurity-related morbidities.^10^

This study aimed to examine the long-term effects of protein-enriched, fortified human milk diets on the growth and neurodevelopment of EPT infants. We hypothesized that infants randomized to receive a higher enteral protein intake between postnatal days 14 and 50 would have a lower incidence of moderate to severe NDI at approximately 2 years CA.

## Methods

### Study design and participants

This was a secondary analysis of a randomized clinical trial in which EPT infants born at 25 to 28 weeks of gestation were included and EPT infants with gastrointestinal malformations, central nervous system malformations, or terminal illness were excluded. During the trial, infants were randomly assigned to receive either fortified milk supplemented with extensively hydrolyzed protein (high protein group) or standard fortified milk (standard protein group) around postnatal day 14.^11^ This approach increased protein content by approximately 0.75 g per 100 mL in the high protein group. Human milk fortification was initiated at ~2 weeks after birth and the study intervention continued until 32 weeks PMA or postnatal day 50, whichever occurred first, for an average duration of 23 days. Participants included infants born at the University of Alabama at Birmingham (UAB) Hospital NICU from 2018 to 2019. The original trial provides a detailed description of the procedures used to screen participants and obtain written parental consent.^11^ Body composition was the primary outcome of the trial. At 36 weeks PMA, body fat percentage did not differ between groups, but fat-free mass (FFM) z-scores were higher in the high protein group. No infants died or developed stage 2 or higher necrotizing enterocolitis (NEC), and four infants experienced grade 3 or higher intraventricular hemorrhage (IVH). There were no significant differences between the high and standard protein groups in the duration of mechanical ventilation or days on supplemental oxygen during the first 28 days after birth. The proportion of infants requiring supplemental oxygen at 28 days was also similar (29% vs. 21%), and none of the infants developed grade 2 or higher bronchopulmonary dysplasia (BPD) by 36 weeks PMA.

### Nutrition interventions

All infants were fed unfortified maternal or donor milk during the first 2 weeks after birth. After postnatal day 14, they were fed maternal or donor milk fortified with a commercially available human milk fortifier intended for preterm infants (Similac Human Milk Fortifier Hydrolyzed Protein Concentrated Liquid, Abbott, Columbus, OH). Infants randomized to the high protein group had a protein supplement (Liquid Protein Fortifier, Abbott, Columbus, OH) added to their feeds the day after the standard bovine fortification was initiated. Technicians in the milk preparation room allocated the study intervention and dispensed feeding syringes with labels that did not show the treatment allocation. After 32 weeks PMA, preterm formula was prescribed until discharge if maternal milk supply was insufficient.

During the trial, both randomization groups initiated enteral feeding on postnatal day 3 and reached enteral feeding volumes greater than 120 ml/kg/day by day 10. At postnatal day 7, enteral protein intake was approximately 1 g/kg/day, and parenteral protein intake averaged 3 g/kg/day. Infants in the high protein group received 0.6 to 1.0 g/kg/day more enteral protein compared to the standard protein group between postnatal day 21 and 32 weeks PMA. Protein-to-energy ratios were also 0.9 to 1.0 g/100 kcal higher in the high protein group. Over the first 28 days after birth, neither daily maternal milk intake (adjusted mean difference: –18 ml/kg; 95% CI: –28 to 65; *p* = 0.43) nor daily maternal-to-donor milk ratios (adjusted mean difference: +0.07; 95% CI: −0.21 to 0.34; *p* = 0.63) differed between groups. There were no significant differences in enteral energy intake between groups. Nutrition plans were guided by weekly multidisciplinary rounds focused on growth goals, but these plans were not dictated by the trial protocol.

### Neurodevelopmental impairment

Moderate to severe NDI was defined as a BSID III or IV cognitive composite score below 85, a Gross Motor Function Classification System (GMFCS) level ≥2, bilateral blindness, or hearing impairment at the routine 2-year follow-up visit. Participants meeting any of these criteria were classified as having moderate to severe NDI. For infants who did not undergo formal neurodevelopmental assessment, physician chart review was conducted to identify potential BSID assessments conducted after the 2-year follow-up visit and rule out moderate to severe NDI based on other routine physician assessments performed at approximately 2 years CA. Infants with a clinical diagnosis of cerebral palsy, developmental delay, blindness, and hearing impairment met criteria for moderate to severe NDI by physician chart review. Participants lost to follow-up without any neurodevelopmental and growth data at approximately 2 years CA were excluded from the analysis. To account for elevated mortality among the most severely impaired infants, the composite outcome of moderate to severe NDI or death was reported.

### Growth

Weight measurements obtained at 18-to-26-months CA were collected through electronic chart review. Weight-for-age z-scores at follow-up were calculated using the World Health Organization Child Growth Standards for infants aged 0 to 36 months based on corrected age rather than chronological age. Weight-for-age z-scores at birth, postnatal day 14, and 36 weeks PMA were calculated using the INTERGROWTH-21^ST^ Standards. Weight-for-age z-scores at each time point were analyzed as continuous variables.

### Covariates

Covariates included gestational age in days and insurance status. Insurance status was used as a proxy variable for socioeconomic status, with participants categorized as having either ‘private insurance’ or ‘public insurance/uninsured’ at baseline.

### Statistical methods

The original trial was powered to detect 3 percentage points difference in body fat percentage using a t-test. Baseline and outcome variables were compared between study groups using Fisher’s exact tests for binary categorical variables and Mann-Whitney U Tests for continuous variables. A repeated-measures linear mixed-effects model was used to examine weight z-score trajectories between the high and standard protein groups across the time-points of birth, postnatal day 14, 36 weeks PMA, and follow-up at 2 years CA. Causal mediation analysis was performed using bootstrapping with 1000 simulations to determine whether change in weight-for-age z-score from birth to follow-up mediates the relationship between study group and NDI.

## Results

Neurodevelopmental outcome data were available for 52 of the 56 infants (92.9%) randomized in the original trial. Of 52 infants with follow-up data, 33 underwent formal BSID assessment (63.5%) and 19 (36.5%) had neurodevelopmental outcomes assessed through physician chart review. The 33 infants who had formal BSID assessments (16 in the high protein group and 17 in the standard protein group) were born at lower gestational ages and had lower birthweights than the 19 infants who were assessed through physician chart review.

Demographic characteristics did not differ significantly between the randomization groups (*n* = 52) [Table 1]. Gestational age (GA) did not differ significantly between randomization groups, with a median GA of 187.5 days (26.8 weeks) in the high protein group and 190.5 days (27.2 weeks) in the standard protein group (*p* = 0.09). Over half of infants were female, 58% were Black, and 75% had public insurance or were uninsured. The median CA at 2-year follow-up among infants assessed for NDI by BSID was 23.8 months in the high protein group and 23.8 months in the standard protein group.

**Table 1:** Baseline Characteristics and Outcomes*.

	High protein group	Standard protein group	*p* value
	(*N* = 28)	(*N* = 24)	
GA at birth in days	187.5 (181–198)	190.5 (186–198)	0.10
Birthweight in grams	890 (708–1154)	910 (747–1118)	0.82
Female Sex	18 (64.3)	13 (54.2)	0.65
Black Race	16 (57.1)	14 (58.3)	1.00
Public insurance	22 (78.6)	17 (70.8)	0.75
CA at BSID assessment in months	23.7 (22.3–24.7)	23.8 (22.5–33.7)	0.39
Moderate to severe NDI or death by either BSID or chart review	12 (42.9)	10 (41.7)	1.00
BSID Cognitive Composite	80 (70–85)	85 (80–85)	0.20
BSID Language Composite	79 (70–86)	77 (72–92)	1.00
BSID Motor Composite	86.5 (82–91)	85 (79–94)	0.74
CA at growth assessment in months**	22.1 (19.9–23.0)	21.9 (20.4–22.4)	0.72
Weight (kg) at 18 to 26 months**	9.9 (8.9–11.4)	10.5 (9.9–11.8)	0.35
z-score	−0.87 (−2.34–0.19)	−0.31 (−1.17–0.34)	0.40
Length (cm) at 18 to 26 months***	81.3 (75.1–83.8)	80.2 (77.2–84.3)	0.77
z-score	−1.02 (−2.77–−0.53)	−0.75 (−1.71–−0.04)	0.46
Head circumference (cm) at 18 to 26 months****	46.2 (44.2–47.8)	47.5 (46.0–48.9)	0.20
z-score	−0.45 (−2.00–0.37)	−0.08 (−1.02–0.75)	0.41
BMI at 18 to 26 months	14.9 (14.5–16.2)	15.9 (15.3–16.9)	0.16
Change in weight z-score from birth to follow-up	−0.71 (−1.41 to −0.03)	−0.05 (−1.04 to 0.60)	0.10
Change in weight z-score from 36 weeks to follow-up	−0.12 (−0.90 to 1.04)	0.66 (−0.30 to 1.82)	0.18

* Categorical and continuous variables are summarized as *n* (%) and median (IQR), respectively.

** *n* = 35; 18 in the high protein group and 17 in the standard protein group.

*** *n* = 30; 15 in the high protein group and 15 in the standard protein group.

**** *n* = 28; 15 in the high protein group and 13 in the standard protein group.

In the high protein group, 16 infants exhibited no/mild NDI, 10 were classified as having moderate to severe NDI and 2 died after discharge. In the standard protein group, 14 infants had no/mild NDI, and 10 had moderate to severe NDI. Fisher’s exact tests revealed no statistically significant difference in the distribution of moderate to severe NDI or death between the two groups (43% vs. 42%; OR = 1.05; 95% CI: 0.35–3.17; *p* = 1.00). Unadjusted logistic regression revealed that the association between randomization group and moderate to severe NDI or death was not significant (OR = 1.05; 95% CI: 0.35–3.20; *p* = 0.93). In an adjusted logistic regression to account for gestational age and type of insurance, randomization group (p = 0.84) was not significantly associated with NDI.

Among the 52 infants with NDI assessments completed by BSID or physician chart review, the 22 who experienced moderate to severe NDI or death had lower median FFM z-scores at 36 weeks PMA ( − 2.1; IQR: −3.2 to −1.3) compared to the 30 without NDI ( − 1.0; IQR: −2.0 to −0.5). This difference approached statistical significance (*p* = 0.05). The linear correlation between FFM z scores at 36 weeks PMA and cognitive scores at 2 years CA was weak and did not reach statistical significance (Pearson correlation coefficient: 0.33; *p* = 0.09).

**Fig. 1:**
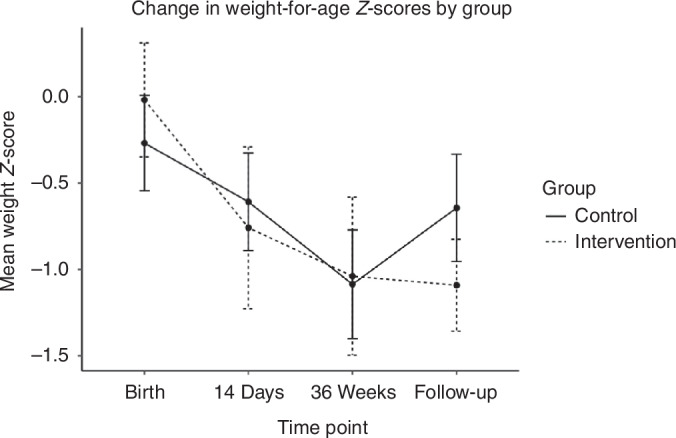
Repeated-measures linear mixed-effects model. Change in Weight-for-age Z-Scores by Group: A linear mixed-effects model was used to assess weight-for-age z-score trajectories between the high and standard protein groups across the time-points of birth, postnatal day 14, 36 weeks PMA, and follow-up at 18 to 26 months CA. The model was fit using restricted maximum likelihood (REML) and incorporated a random intercept for study participant to account for repeated measures.

At 18 to 26 months CA, weight measurements were available for 35 infants (67%), length measurements were available for 30 infants (58%), and head circumference measurements were available for 28 infants (54%). Infants with weight measurements (*n* = 35) had similar GA, birthweight, and in-hospital growth parameters up to 36 weeks PMA than those infants without these measurements (*n* = 17). Neither weight, length, head circumference nor the associated z-scores differed significantly between the study groups at follow-up. A linear mixed-effects model was used to assess weight-for-age z-score trajectories between the high and standard protein groups across the time-points of birth, postnatal day 14, 36 weeks PMA, and follow-up at 18 to 26 months CA [Fig. 1]. There were no significant differences in weight z-scores between the 18 infants in the high protein group and the 17 in the standard protein group.

Causal mediation analysis was performed using bootstrapping with 1000 simulations to examine whether weight-for-age z-score at follow-up mediates the relationship between randomization group and NDI. The average direct effect of randomization group on NDI (*p* = 0.820, 95% CI: −0.31 to 0.36), mediation effect (*p* = 0.440, 95% CI: −0.06 to 0.17), and the proportion mediated (*p* = 0.750, 95% CI: −3.26 to 4.65) were not statistically significant.

## Discussion

The present secondary analysis examined if the improvements in weight and FFM-for-age z-scores at 36 weeks PMA observed in the original trial were associated with sustained growth benefits at 2 years CA and a reduction in the incidence of moderate to severe NDI. Our findings indicate that high-protein versus standard-protein human milk diets in the NICU did not significantly impact the incidence of moderate to severe NDI or death at two years CA. Infants in the high protein group demonstrated more negative growth trajectories from birth to 2 years of CA than those in the standard protein group, but these differences did not reach statistical significance. These findings suggest that while supplemental protein may have positive short-term effects on weight and FFM-for-age z-scores at 36 weeks PMA, these benefits do not persist after EPT infants transition to post-discharge diets.

Preterm birth disrupts the critical period of brain development and maturation that occurs between 24 and 40 weeks of gestation, increasing the risk of severe morbidities and developmental delays. Given the vulnerability of EPT infants, identifying modifiable protective factors such as increased protein intake could inform nutritional guidelines and improve long-term outcomes. Observational studies have linked early enteral protein intake with increased brain volume^12^ and larger linear measurements of key brain structures, such as the corpus callosum, caudate head, and cerebellum, at postnatal day 28 in preterm infants.^13^ Additionally, an association has been demonstrated between postnatal weight gain and increased volumes of the cerebellum, basal ganglia, thalami, and cortical gray matter in very preterm infants (<31 weeks of gestation).^14^ Notably, enteral protein intake has been associated with greater cerebellar and cortical gray matter volume, suggesting that early nutritional interventions could play a role in optimizing neurodevelopmental outcomes.^15^

While previous studies have demonstrated the benefits of protein supplementation on brain^12^ and somatic growth during hospitalization, the long-term effects remain uncertain. Previous meta-analyses of randomized clinical trials comparing high versus standard protein failed to identify trials that reported long-term growth and neurodevelopmental outcomes.^16,17^ Our findings are consistent with prior research indicating that high-protein intake in the NICU promotes early growth but may not lead to sustained benefits post-discharge. The decline in weight-for-age z-scores observed in the high protein group likely represents a regression to baseline growth trajectories rather than a detrimental effect of supplementation. These findings highlight the need for long-term, well-controlled studies to determine if early nutritional interventions can have a lasting impact on growth and neurodevelopment in preterm infants.^11^

### Strengths

A major strength of this study is its randomized, masked design, which minimizes biases and ensures that observed differences in outcomes are attributable to the intervention rather than confounding factors. Additionally, this study extends beyond in-hospital outcomes by including post-discharge assessments at approximately two years CA, a significant advantage over previous neonatal nutrition studies that focus primarily on short-term metrics.

### Limitations

Several limitations must be acknowledged. The primary limitation of this study was the smaller-than-intended sample size, likely influenced by the COVID-19 pandemic’s impact on routine medical care. This limited our statistical power and restricted our ability to conduct stratified subgroup analyses. Another challenge was attrition bias, as parents of infants with developmental concerns were more likely to return for follow-up visits than those with typically developing children. Although we attempted to mitigate this bias through physician chart reviews of infants who presented for routine outpatient care, this method lacks standardization and may impact the generalizability of our findings. Furthermore, this study did not assess whether the initiation of protein enrichment before achieving full enteral nutrition could mitigate nutritional deficits and improve long-term outcomes. Additionally, body composition measurements were not performed at the two-year follow-up visit, making it difficult to determine whether the observed declines in weight-for-age z-scores post-discharge were due to reduced FFM accretion or a protective effect against excessive fat mass gain.

## Conclusions

This secondary analysis did not identify a significant impact of high protein supplementation after achieving full enteral nutrition on weight-for-age z-scores or survival without moderate to severe NDI in EPT infants. These findings underscore the need to bridge early nutritional interventions with sustained post-discharge benefits. Future research should explore strategies to maintain the positive effects of early protein supplementation beyond hospital discharge and optimize long-term growth and neurodevelopmental outcomes in this high-risk population.

## Data Availability

The datasets generated during and/or analyzed during the current study are available from the corresponding author on reasonable request.
